# Epidemiology and Clinical Features of Pulmonary Nontuberculous Mycobacteriosis in Nagasaki, Japan

**DOI:** 10.1371/journal.pone.0128304

**Published:** 2015-05-28

**Authors:** Shotaro Ide, Shigeki Nakamura, Yoshihiro Yamamoto, Yoshihisa Kohno, Yuichi Fukuda, Hideki Ikeda, Eisuke Sasaki, Katsunori Yanagihara, Yasuhito Higashiyama, Kohji Hashiguchi, Yoji Futsuki, Yuichi Inoue, Kiyoyasu Fukushima, Naofumi Suyama, Shigeru Kohno

**Affiliations:** 1 Department of Respiratory Diseases, Nagasaki University Graduate School of Biomedical Sciences, Nagasaki, Japan; 2 Unit of Molecular Microbiology and Immunology, Nagasaki University Graduate School of Biomedical Sciences, Nagasaki, Japan; 3 Department of Laboratory Medicine, Nagasaki University Hospital, Nagasaki, Japan; 4 Japanese Red Cross Nagasaki Genbaku Hospital, Nagasaki, Japan; 5 Nagasaki Municipal Hospital, Nagasaki, Japan; 6 Isahaya Health Insurance General Hospital, Isahaya, Japan; 7 Japanese Red Cross Nagasaki Genbaku Isahaya Hospital, Isahaya, Japan; 8 Sasebo City General Hospital, Sasebo, Japan; 9 Hokusho Central Hospital, Sasebo, Japan; 10 National Hospital Organization Nagasaki Medical Center, Omura, Japan; 11 Izumikawa Hospital, Minami-Shimabara, Japan; 12 Nagasaki Goto Central Hospital, Goto, Japan; 13 Department of Chemotherapy and Mycoses, National Institute of Infectious Diseases, Shinjuku-ku, Tokyo, Japan; 14 Department of Clinical Infectious Diseases, Toyama University Graduate School of Medicine and Pharmaceutical Sciences, Toyama, Japan; Cambridge University, UNITED KINGDOM

## Abstract

**Background and Objectives:**

Recent reports indicate that the incidence of nontuberculous mycobacterial-lung disease (NTM-LD) is increasing. This study aimed to investigate the epidemiology and clinical features of NTM-LD patients in Nagasaki prefecture, Japan to identify the negative prognostic factors for NTM-LD in Japan.

**Methods:**

The medical records of patients newly diagnosed with NTM-LD in eleven hospitals in Nagasaki prefecture between January 2001 and February 2010 were reviewed. Data regarding the annual population of each region and the incidence of all forms of tuberculosis were collected to assess geographic variations in NTM-LD incidence, isolates, and radiological features.

**Results:**

A total 975 patients were diagnosed with NTM-LD. The incidence increased over the study period and reached 11.0 and 10.1 per 100,000 population in 2008 and 2009, respectively. *M*. *intracellulare* was the most common pathogen in the southern region, and *M*. *avium* most common in other regions. The most common radiographic pattern was the nodular-bronchiectatic pattern. Age >60 years, body mass index <18.5 kg/m^2^, underlying lung disease, and cavitary pattern were the negative prognostic factors at the 1-year follow-up.

**Conclusions:**

The incidence of NTM-LD has been increasing in Nagasaki prefecture. The isolates and radiographic features of patients vary markedly by region.

## Introduction

Nontuberculous mycobacteria (NTM) are ubiquitous organisms that cause diverse types of infectious disease in human organs, including lung, lymphatic, disseminated, skin, soft tissue, and bone disease [1,2]. Several reports have suggested that the incidence of nontuberculous mycobacterial lung disease (NTM-LD) has recently been increasing [1,3–6]. Although determining the precise incidence and prevalence of NTM-LD is difficult, the incidence in Japan was estimated at 5.9 per 100,000 population in 2005 [7], which was higher than that of other countries in the same year [4,6,8]. Although *Mycobacterium* species vary markedly by geographic region, *Mycobacterium avium-intracellulare* complex (MAC) has been reported as the dominant species in NTM-LD [5,9]. In Japan, *M*. *avium* is most commonly isolated from the respiratory tract, followed by *M*. *intracellulare*. Nevertheless, the species involved in NTM-LD has been found to vary even within Japan [7].

Previous studies reported the several risk factors for NTM infection such as gastroesophageal reflux disease, chronic obstructive pulmonary disease (COPD), cystic fibrosis, history of tuberculosis and immune defects or diseases such as HIV infection, corticosteroid use [1,10]. Negative prognostic factors for HIV-negative patients with NTM-LD are high comorbidity level, advanced age, male sex, fibrocavitary disease, body mass index (BMI) <18.5 kg/m^2^, and anemia [11,12]. This study aimed to investigate the epidemiology and clinical features of NTM-LD patients in Nagasaki prefecture to identify the risk factors and negative prognostic factors in Japan.

## Patients and Methods

### Participants

Nagasaki prefecture provides approximately 14,700 hospital beds for the medical care of a population of approximately 1,500,000. The participants were selected by review of the microbiological database at 11 hospitals (total 4,218 beds total) in the prefecture between January 2001 and February 2010. The participating facilities were Nagasaki University Hospital (862 beds), Nagasaki Municipal Hospital (414 beds), Nagasaki Municipal Medical Center (217 beds), Japanese Red Cross Nagasaki Genbaku Hospital (360 beds), Japanese Red Cross Nagasaki Genbaku Isahaya Hospital (140 beds), Isahaya Health Insurance General Hospital (333 beds), National Hospital Organization Nagasaki Medical Center (650 beds), Izumikawa Hospital (120 beds), Sasebo City General Hospital (594 beds), Hokusho Central Hospital (224 beds), and Nagasaki Goto Central Hospital (304 beds). The sputum, bronchial wash and tissue samples were cultured using Ogawa egg-based media and/or a Mycobacteria Growth Indicator Tube (MGIT; Becton Dickinson Microbiology Systems, Sparks, MD, USA). Isolated specimens were identified using polymerase chain reaction (PCR) or DNA-DNA hybridization (DDH) for *M*. *avium* and *M*. *intracellulare*; DDH for *M*. *kansasii*, *M*. *abscessus*, *M*. *kansasii*, *M*. *gordonae*, *M*. *chelonae*, *M*. *szulgai*, *M*. *simiae*, *M*. *peregrinum*, *M*. *scrofulaceum* and *M*. *terrae*; and 16S ribosomal RNA sequencing for *M*. *shimoidei* [13,14]. Patients with at least two separate culture-positive sputum samples or one culture-positive bronchial washing or lung tissue sample during the study period were included. Patients with a single positive sputum culture or PCR positive-culture negative samples were excluded.

### Diagnosis of NTM-LD

Diagnosis was performed by specialists in infectious disease and/or respiratory medicine according to 2007 American Thoracic Society (ATS)/Infectious Diseases Society of America (IDSA) criteria [1]. In accordance with the criteria, patients with suspected infection with contaminated environmental *Mycobacterium* species were excluded from study participation. Patients who had been diagnosed with NTM-LD prior to the current study were also excluded.

### Epidemiological Study

**Fig 1:**
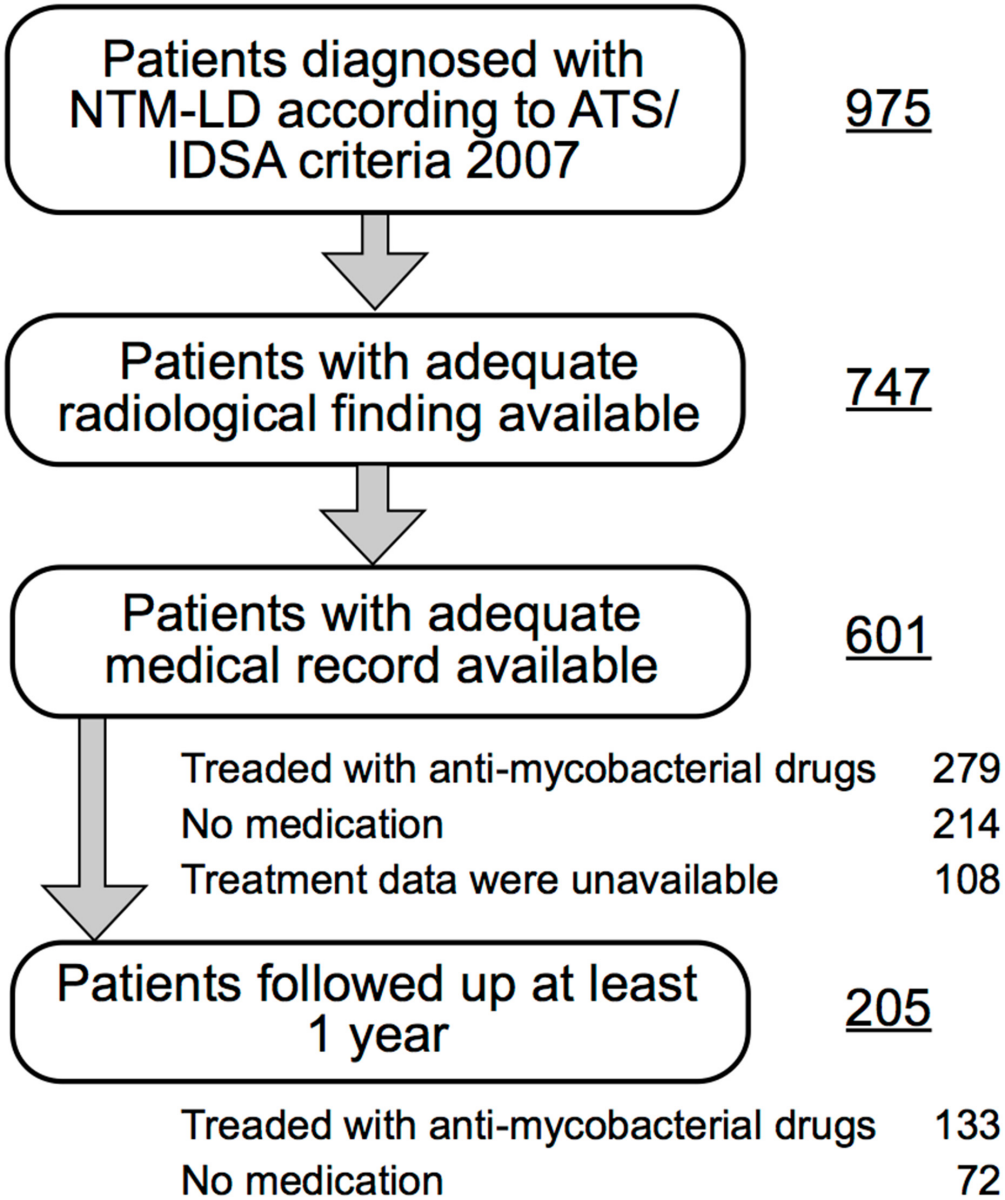
Flowchart of patient selection.

Among all the patients who had presented at the 11 hospitals during the study period, 975 had been diagnosed with NTM-LD according to the 2007 ATS/IDSA criteria. After dividing the prefecture into five regions on the basis of administrative boundaries and medical care zones, the patients were classified into one region based on the residence listed in their medical records. To estimate the annual incidence of NTM-LD in each region, data regarding the annual population of each region were collected from the Nagasaki prefectural government, and data regarding the incidence of all forms of tuberculosis collected from the healthcare center of each region. In the analysis of the radiological features, 228 patients were excluded due to lack of accurate information about chest computed tomography (CT) scan (Fig 1). Based on the review of the results of chest CT scan, the remaining 747 patients were classified into four patterns according to their radiological characteristics: the nodular-bronchiectatic (NB), cavitary (CAV), hypersensitivity pneumonitis-like, or unclassifiable pattern. The NB pattern and CAV pattern were defined by chest CT scan showing multifocal bronchiectasis with multiple small nodules or cavitary opacities, respectively. If a patient had the characteristics of both the NB and CAV patterns, the patient was classified in accordance with the dominant pattern. Using these data, geographic variations in NTM-LD incidence, isolates, and radiological features were assessed.

### Patient Characteristics and Prognostic Factors

Of the 975 patients, 374 were excluded from analysis due to lack of sufficient clinical information in their medical records (Fig 1). The clinical course of the disease was assessed after 1 year of diagnosis. Patient outcome was evaluated by review of (1) clinical, (2) radiological and (3) microbiological responses. Clinical response was defined as resolution of symptoms such as fever, weight loss, cough, sputum, hemoptysis and general fatigue. Radiological response was defined as the clearing or improvement of opacity on chest radiographs/CT. Microbiological response was determined by culturing of sputum. Patients who had shown improvement or no change in the 3 factors and had experienced no deterioration were defined as having achieved “stability”. Patients who had not met the criteria for “stability” definition and had subsequently died from all causes were classified as having experienced “deterioration”.

### Statistical Analysis

The data are presented as mean ± SD values. Statistical significance was evaluated using the chi-square test or the two-tailed Mann-Whitney test, with the level of significance set at P < 0.05. All analyses were performed with GraphPad Prism 5.0 for Macintosh (GraphPad Software, La Jolla, CA, USA).

### Ethics Statement

This study was approved by the Ethical Committee of the Nagasaki University Hospital. As this study was retrospective, and data were analyzed anonymously, the need to obtain patient consent was waived by the committee.

## Results

**Fig 2:**
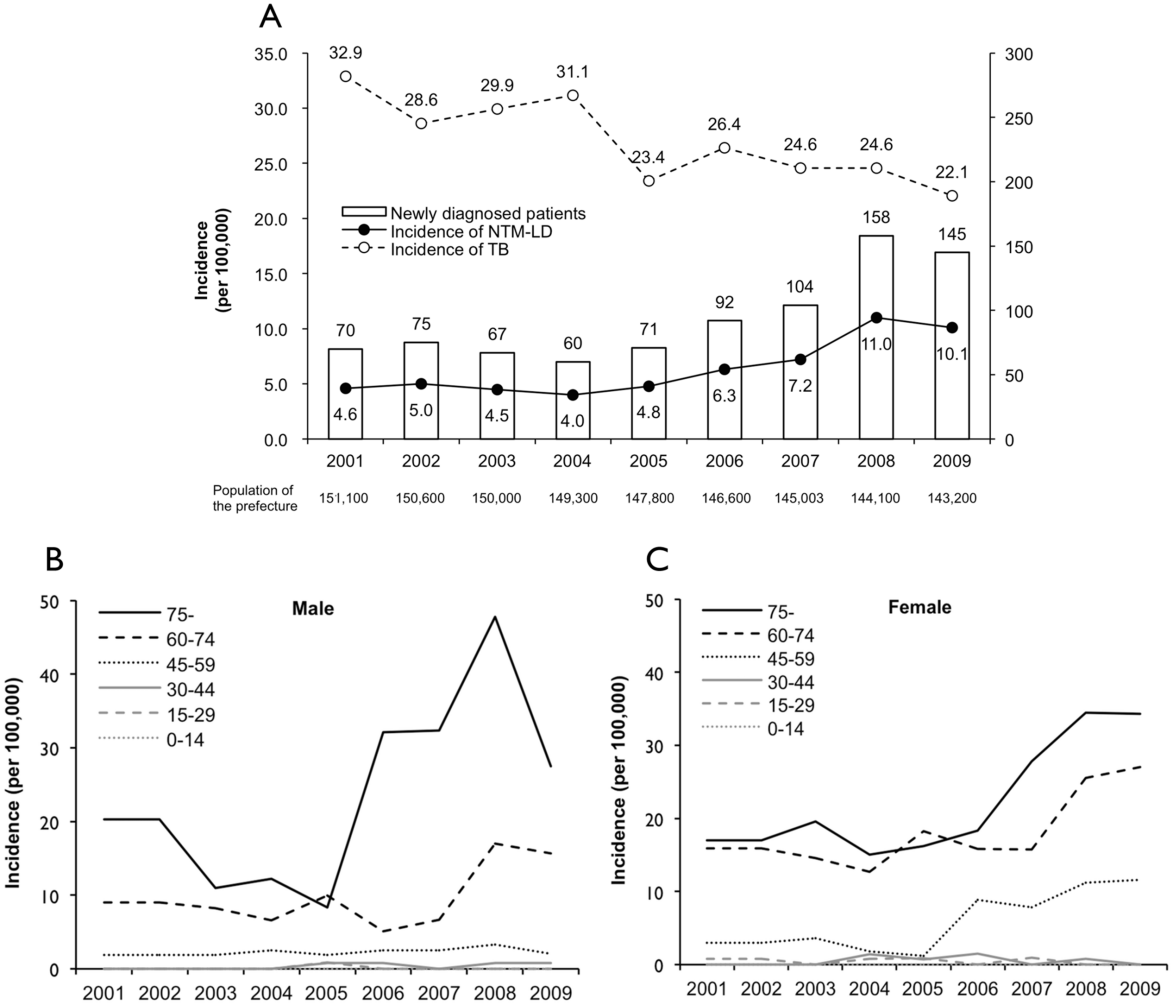
Annual incidence of NTM-LD between 2001 and 2009 in Nagasaki, Japan. (A) Incidence of NTM-LD and all types of tuberculosis in all patients. (B) Incidence in male patients. (C) Incidence in female patients.

Fig 2A shows the numbers of NTM-LD patients newly diagnosed each year between 2001 and 2009, the population of Nagasaki prefecture, the incidence of all tuberculosis cases, and the estimated incidence of NTM-LD between 2001 and 2009. As can be observed, 975 patients were newly diagnosed with NTM-LD. Regarding the incidence by year, the NTM-LD incidence gradually increased over the entire study period and especially in 2008 and 2009, when it reached 11.0 and 10.1 per 100,000 population, respectively. The incidence was the highest in the southeastern region of the prefecture, reaching 18.2 and 21.2 per 100,000 population in 2008 and 2009, respectively. Regarding the incidence by age, men and women >60 years accounted for 83.8% of all patients, and the greatest increase in incidence occurred in men and women >60 years and in women 45–59 years (Fig 2B and 2C).

**Fig 3:**
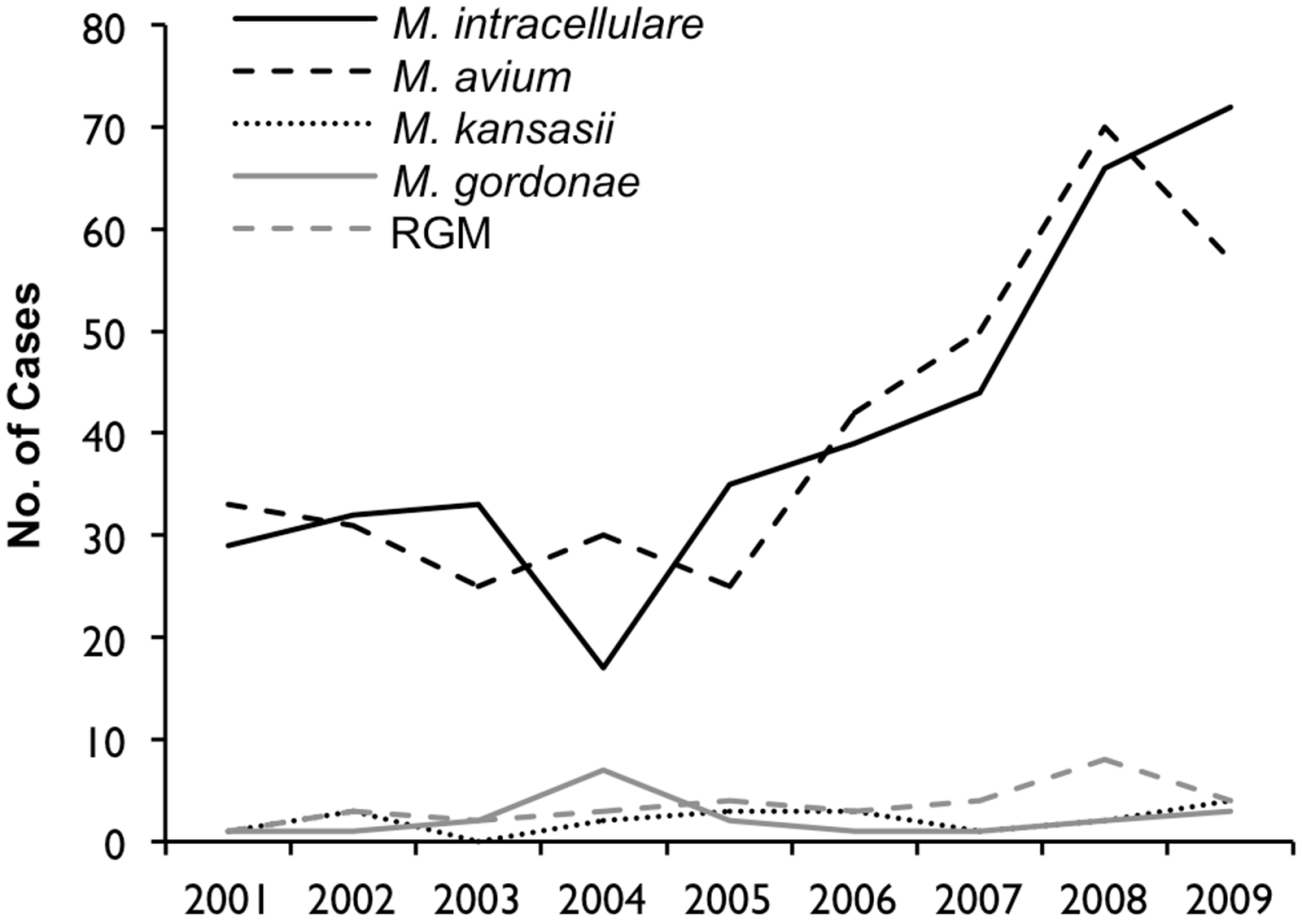
Isolates detected in NTM-LD patients between 2001 and 2009 in Nagasaki, Japan. RGM, rapid growing mycobacteria.

**Fig 4:**
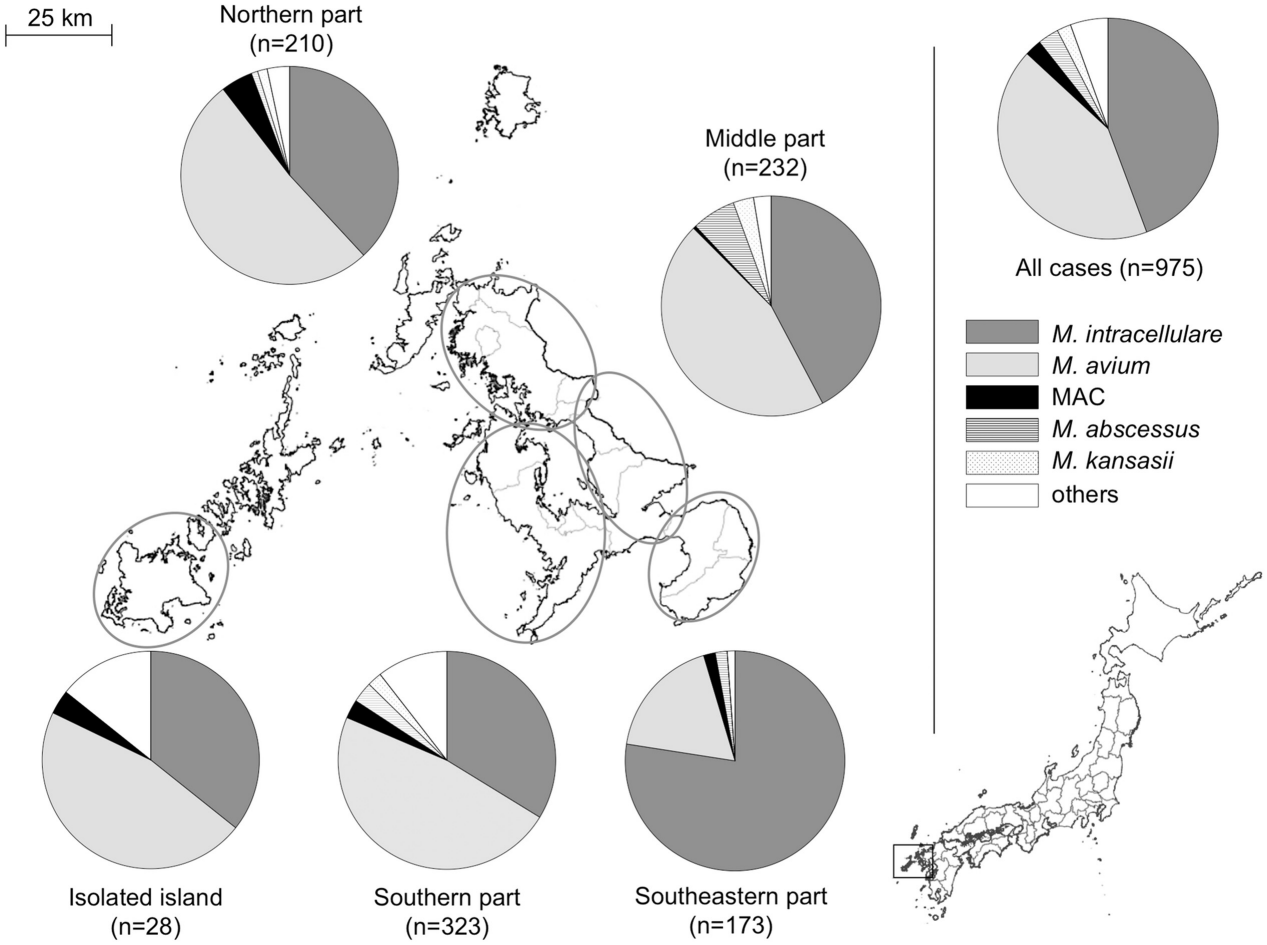
Geographic variation in isolates detected in NTM-LD patients. Nine patients were residents of another prefecture. Map was obtained from the Geospatial Information Authority of Japan (http://www.gsi.go.jp/ENGLISH/index.html). MAC, *Mycobacterium avium-intracellulare* complex.

**Fig 5:**
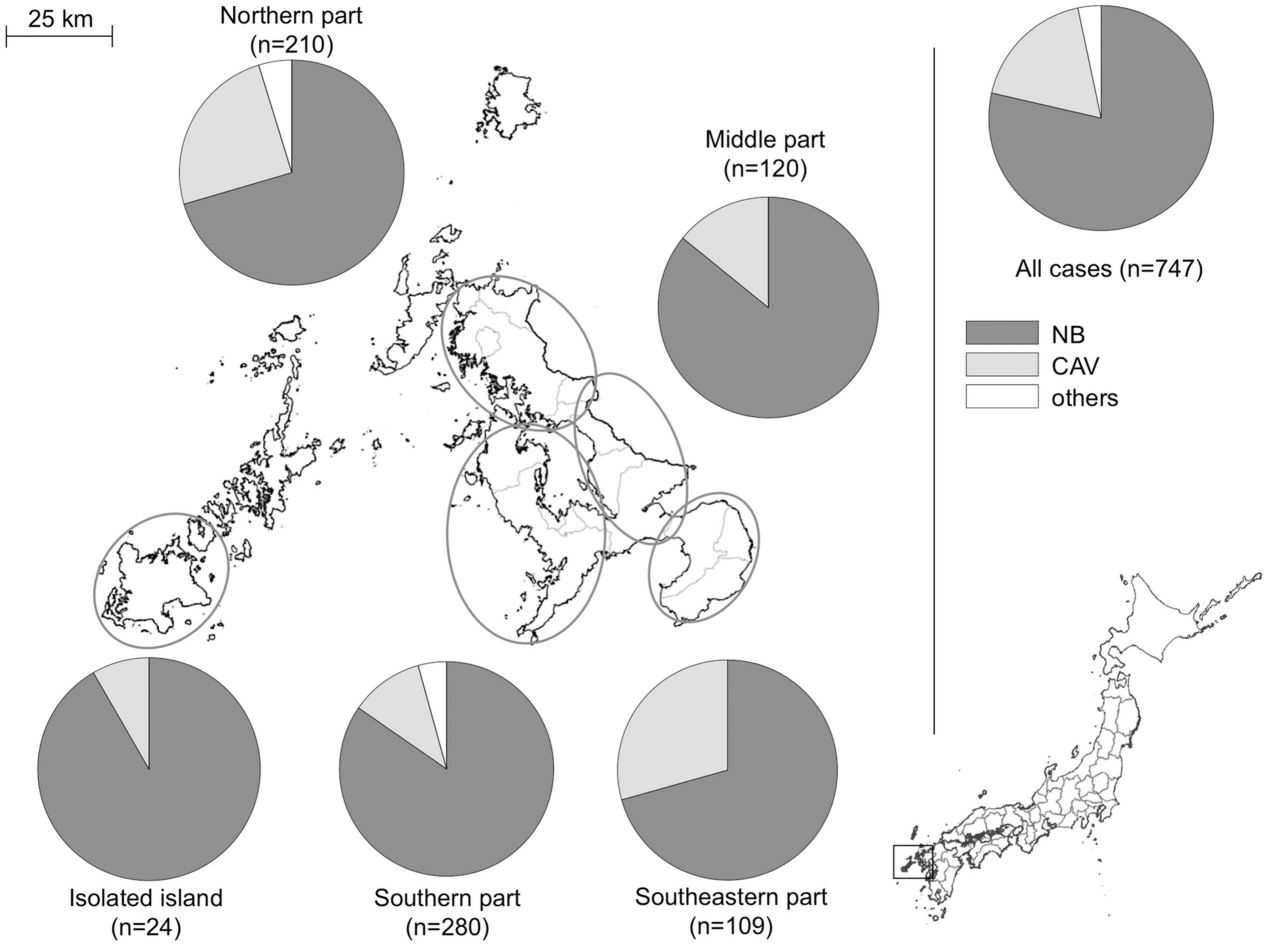
Geographic variation in radiographic patterns in NTM-LD patients. Four patients were residents of another prefecture. NB, nodular-bronchiectatic pattern; CAV, cavitary pattern.

Table 1 shows that the most frequently identified pathogen was *M*. *intracellulare* (n = 432, 44.3%), followed by *M*. *avium* (n = 415, 42.6%), *M*. *abscessus* (n = 30, 3.1%), *M*. *avium-intracellulare* complex (n = 24, 2.5%), *M*. *kansasii* (n = 20, 2.1%), *M*. *gordonae* (n = 20, 2.1%), and other pathogens (n = 34, 3.5%). Although the incidence of infection with *M*. *avium* and *M*. *intracellulare* particularly increased over the study period (Fig 3), the distribution of the isolates varied throughout the prefecture, with *M*. *intracellulare* accounting for 77.5% of all the isolates detected in the southern region (Fig 4). The most common radiographic pattern was the NB pattern, which was observed in 591 cases (79.1%), followed by the CAV pattern, which as observed in 134 cases (17.9%). No patients were found to have the hypersensitivity-like pattern. The percentage of patients with each pattern was found to vary by region, especially in the southern region, where CAV pattern was observed in 29.4% of the patients (Fig 5).

**Table 1:** Isolates detected in 975 NTM-LD patients.

Isolate	Number (%)
*M*. *intracellulare*	432 (44.3)
*M*. *avium*	415 (42.6)
*M*. *abscessus*	30 (3.1)
MAC	24 (2.5)
*M*. *kansasii*	20 (2.1)
*M*. *gordonae*	20 (2.1)
*M*. *chelonae*	7 (0.7)
*M*. *fortuitum*	3 (0.3)
*M*. *szulgai*	3 (0.3)
*M*. *simiae*	2 (0.2)
*M*. *peregrinum*	2 (0.2)
*M*. *scrofulaceum*	2 (0.2)
*M*. *terrae*	2 (0.2)
*M*. *shimoidei* *	1 (0.1)
PMI except for MAC	7 (0.7)
Not identified	5 (0.5)

MAC, *Mycobacterium avium-intracellulare* complex; PMI, polymicrobial infection

*Identified using 16S rRNA.

Of the 975 patients, the clinical information of 601 patients with a mean age of 71.2 ± 11.2 years was analyzed. Among these, 182 (30.3%) patients were male with a mean age of 73.0 ± 10.9 years and 419 (69.7%) female with a mean age of 70.5 ± 11.2 years. Regarding disease state, the underlying respiratory disease was identified in 89 (48.9%) male and 91 (21.7%) female patients (Table 2) and systemic complications (diabetes, collagen disease, malignant disease other than lung cancer, chronic kidney disease, and chronic hepatic disease) in 52 (28.6%) male and 87 (20.8%) female patients. The underlying disease in 53 (8.9%) patients was treated by the administration of corticosteroids and/or immunosuppressants.

**Table 2:** Underlying respiratory diseases of NTM-LD.

	Total		Male		Female		P value
	601	(%)	182	(%)	419	(%)	
Underlying lung disease	189	(31.4)	90	(49.5)	99	(23.4)	<0.0001
History of tuberculosis	81	(13.5)	35	(19.2)	46	(11.0)	0.0065
COPD	35	(5.8)	22	(12.1)	13	(3.1)	<0.0001
Lung cancer	33	(5.5)	17	(9.3)	16	(3.8)	0.0063
Interstitial lung disease	24	(4.0)	13	(7.1)	11	(2.6)	0.0094
Fungal infection	15	(2.5)	9	(4.9)	6	(1.4)	0.0112
History of lung surgery	13	(2.2)	9	(4.9)	4	(1.0)	0.002
Bronchial asthma	11	(1.8)	4	(2.2)	7	(1.7)	0.6578
Chronic bronchitis	9	(1.5)	5	(2.7)	4	(1.0)	0.0964
Silicosis	8	(1.3)	8	(4.4)	0	(0.0)	<0.0001
Bronchiectasis	2	(0.3)	1	(0.5)	1	(0.2)	0.5433
Pulmonary sarcoidosis	2	(0.3)	1	(0.5)	1	(0.2)	0.5433

COPD, Chronic Obstructive Pulmonary Disease.

As observed in Table 3, which shows the causes of immunodeficiency, no patient was infected with HIV. The NB pattern was the most common radiologic pattern, as observed in 479 (79.7%) of the 601 patients, followed by the CAV pattern in 94 (15.6%), and an unclassifiable pattern in 28 (4.7%). The number of patients with the NB pattern increased over the study period, whereas the number with the CAV pattern remained unchanged. Regarding treatment, 279 (46.4%) patients had been treated by the administration of 1–5 anti-mycobacterial drugs within 3 months after diagnosis for a mean duration of 14.8 months, 214 (33.9%) patients had been observed clinically without being administered medication for 1 year, and treatment data were unavailable for 108 (18.0%) after 1 year (Fig 1). The analysis of prognostic factors was performed with the 205 patients who had been followed up for at least 1 year after diagnosis. Of the 86 patients (42.0%) who had improved, 75 (87.2%) had been treated with anti-mycobacterial drugs and 11 (12.8%) had been treated with no medication. Poor prognostic factors were found to be age >60 years, BMI<18.5 kg/m^2^, underlying lung disease, and radiographic CAV pattern (Table 4).

**Table 3:** Causes of immunodeficiency.

	N = 601 (%)	
Steroid administration	31	(5.2)
Methotrexate (MTX) administration	4	(0.7)
Immunosuppressant administration except MTX	8	(1.3)
Steroid and immunosuppressant administration	10	(1.7)
Diabetes	54	(9.0)
Collagen disease	30	(5.0)
Malignant disease	44	(7.3)
Chronic kidney disease	9	(1.5)
Chronic hepatic disease	22	(3.7)

**Table 4:** Characteristics and 1-year prognosis of NTM-LD patients.

	Total (%)	Stability (%)	Deterioration (%)	P value
No. of patients	204	159 (77.9)	45 (22.1)	
Age	69.9 ± 11.6	69.2 ± 11.0	73.3 ± 11.3	0.00315
Male/female	61/143	46/113	15/30	0.3243
BMI < 18.5 kg/m^2^	48 (23.5)	33 (20.8)	15 (33.3)	0.0355
CRP level (mg/dL)	0.85 ± 2.13	0.52 ± 1.21	1.49 ± 3.08	0.1431
Smoking pack-years ≥ 20	18 (8.8)	12 (7.5)	6 (13.3)	0.0582
CAM dose (mg)	565.2 ± 214.7	573.5 ± 192.0	542.9 ± 198.9	0.6369
Underlying lung disease*	65 (31.9)	45 (28.3)	20 (44.4)	0.0089
CAV	25 (12.3)	15 (9.4)	10 (22.2)	0.006

BMI, body mass index; CRP, C-reactive protein; CAM, clarithromycin; CAV, cavitary pattern on chest radiographs.

*Includes history of tuberculosis, chronic obstructive pulmonary disease, lung cancer, interstitial lung disease, asthma, and silicosis.

## Discussion

NTM-LD has been increasing over the past few decades in many areas of the world [1,3–5,15]. The results of the present study revealed that the annual incidence of NTM-LD in Nagasaki prefecture, Japan exceeded 10.0 per 100,000 population in 2008 and 2009. This incidence is much higher than that of other developed countries over the past decade, which has been reported as 2.7–5.6/100,000 in the United States [4,16,17], 0.72–0.74/100,000 in France [18], 0.9–2.9/100,000 in the United Kingdom [19], 2.2–3.2/100,000 in Australia [6], 1.06–2.00/100,000 in Taiwan [8], and 1.08/100,000 in Denmark. The dynamics of NTM-LD may be affected by various factors, including behavioral [12], cultural, genetic, environmental characteristics [20], soil characteristics, income levels, and population densities [21,22]. In this study, men and women >60 years old accounted for 83.8% of all participants, and the proportion of this age group increased over the study period. This finding and those of previous reports [6,12,19] suggest that the increasing incidence of NTM-LD could be associated with the aging of society and the increasing number of patients with comorbidities. In addition, previous reports indicate that cross-immunity and the protection provided by infection with *M*. *tuberculosis* and use of the bacillus Calmette-Guerin (BCG) vaccine is associated with NTM infection [15,23–25]. Our study also showed that the decreasing rate of tuberculosis is inversely associated with the increase in NTM-LD. However, cross-immunity might not be a key component, since almost all Japanese have been vaccinated with BCG for over half a century, and the southern prefecture, which was found to have a high incidence of NTM-LD in this study, has been a moderately endemic area of tuberculosis (a constant incidence of 28.9–40.4/100,000) over the past decade.

*Mycobacterium* species are ubiquitous organisms present in water, soil, and room dust [26]. In this study, *M*. *avium* was the most commonly detected species in most regions of Nagasaki prefecture, although *M*. *intracellulare* was the most commonly detected in the southern region. This variation may be related to exposure to certain substances present in the soil that have been associated with NTM infection [27,28]. In Australia, Chou et al. reported that the presence of *M*. *intracellulare* infection was correlated with a shallower soil depth and agricultural activities that lead to soil disturbance [22]. As the southern region of Nagasaki prefecture is an agricultural zone with soil well drained by an active volcano, in which the percentage of cultivated area to total area is twice as high as the average of the prefecture (26.5% vs. 12.3%), the soil and agricultural activities performed within it might have led to differences in the dominant isolate and high incidence in this region compared to that in other regions. Additionally, in the southern region, the frequency of radiographic CAV pattern was higher than other areas. As described above, high prevalence of tuberculosis might have led to high rate of CAV pattern.

In general, effective treatment of NTM-LD requires long-term treatment with multiple anti-mycobacterial drugs. ATS/IDSA guidelines recommend that patients with NTM-LD should be treated until negative cultures are obtained for at least 1 year [1]. In addition to sputum culture, assessment of symptom subscore [29], clinical symptoms [30], and radiological findings [30,31] are useful in predicting clinical response. As short-term response to initial treatment has been found to reflect long-term outcome [32], this study evaluated 1-year response by assessment of clinical symptoms, radiological findings, and microbiological responses. The factors of age >60 years, BMI<18.5 kg/m^2^, underlying lung disease, and radiographic CAV pattern were found to be poor prognostic factors, which accords with the findings of Hayashi et al. [11], who found advanced age, systemic and/or respiratory comorbidities, non-NB radiographic features, BMI<18.5 kg/m^2^, anemia, hypoalbuminemia, and erythrocyte sedimentation rate ≥50 mm/h to be negative prognostic factors for all-cause mortality of HIV-negative NTM-LD patients. It also accords with those of Andréjak et al. [12], who found high comorbidity level, age >65 years, and male sex to be predictors of all-cause mortality for NTM-LD patients. Although it remains unclear whether treatment of NTM-LD should be started immediately after diagnosis or be delayed, these findings may be helpful in determining when to start treatment.

This study faced several limitations that should be considered when reviewing the findings. Although a relatively large number of hospitals (11 hospitals) in Nagasaki prefecture were examined, not all NTM-LD cases in Nagasaki could be investigated. Second, the patients whose sputum culture was NTM positive only once were excluded from further analysis. These limitations might have led to underestimation of the incidence of NTM-LD. Third, the long-term prognosis is not shown in this retrospective study since we could not obtain the sufficient data. Additionally, selection bias may have been a factor in selecting the duration of treatment and follow-up, specifically the selection of relatively brief treatment and follow-up periods. However, as described above, short-term response for initial treatment has been reported to reflect long-term outcome, so we believe that this study of prognostic factors 1 year after diagnosis provides useful information.

In conclusion, the incidence of NTM-LD has been increasing over the past decade in Nagasaki, Japan, a prefecture in which the isolates and radiographic patterns have varied markedly by region. Among all possible factors, older age, BMI <18.5 kg/m^2^, underlying lung disease, and radiographic CAV pattern appear to be negative prognostic factors in the deterioration of patients with the disease.
